# Preparation and characterization of nano MnO-CaLs as a green catalyst for oxidation of styrene

**DOI:** 10.3906/kim-2101-26

**Published:** 2021-08-16

**Authors:** Mahdi ARIAN, Hoda MOLLABAGHER, Salman TAHERI, Ali ZAMANIAN, Seyed Amir Hossein Seyed MOUSAVI

**Affiliations:** 1 Biomaterials Research Group, Nanotechnology and Advanced Materials Department, Materials and Energy Research Center (MERC), Tehran, Iran; 2 Chemistry and Chemical Engineering Research Center of Iran (CCERCI), Tehran, Iran; 3 Department of Process Engineering, Faculty of Chemical Engineering, Tarbiat Modares University, Tehran, Iran

**Keywords:** Epoxidation, nanocatalyst, MnO, calcium lignosulfonates, styrene

## Abstract

Hydrophilic nano MnO is shown to have significant stability in aqueous media for oxidation of styrene. Different catalysts have been used to synthesis styrene oxide, but MnO-CaL is considered the efficient and selective catalyst to produce styrene oxide. In general, this paper reported especial strategy for synthesis of novel nano MnO that stabilized with oleic acid in chloroform and changing nature of its stabilizer by exchanging oleic acid with lignosulfunate and displays its catalytic activity towards selective oxidation of styrene. The catalyst has shown good selectivity in oxidation of styrene by changing temperature. Finding the optimal conditions for reaction and determining the best time and temperature for achieving the ideal product and reducing the side products are among the issues discussed in this article. MnO-CaLs leads to selective oxidation of styrene to styrene epoxide at low temperature. By increasing the temperatures, benzaldehyde and partially 2-phenyl acetaldehyde are also produced as by-products. Furthermore, the nano catalyst could be recycled several times without any clear changing in activity, which makes nano catalyst economic and environmentally friendly.

## 1. Introduction

Epoxides are a main class of industrial chemical compound that have been generally used as chemical intermediates in various fields such as agriculture and pharmaceutical science [1]. Generally, epoxides are produced from epoxidation of alkene [2,3], and in this respect, peracids are used in the direct epoxidation of alkenes to produce the related epoxides with producing large amounts of wastes. Synthesis of heterocyclic compounds are interested by chemists due to its pharmaceutical and agricultural application [4,5,6]. Numerous approaches for styrene epoxidation were addressed [7,8], and in view of both economic and environmental aspects, hydrogen peroxide is a suitable oxidant [9]. There are many advantages in applying hydrogen peroxide as the oxidant as it is environmentally friendly, a safe byproduct, and has a low cost [10].

Due to the importance of catalytic oxidation production of styrene-epoxide from styrene in the biological environment and in the industrial reactions, the consideration of high-efficiency and short-term efficient methods has been taken into consideration by chemists. On the other hand, transition metal oxides show good catalytic activity and selectivity and, therefore, are widely applied as heterogeneous catalysts in various chemical reactions due to their benefits over homogeneous catalysts [11] such as providing easy separation, biocompatibility selectivity, and recyclability [12,13,14]. Since energy consumption has become one of the major concerns of the world today, efforts should be made to reduce energy consumption in chemical processes [15]. It should be noted that one of the main roles of catalysts is to reduce energy consumption in response by reducing activation energy.

Among transition metal oxide, manganese oxides have a wide application in catalytic reaction due to its special structure of crystals and chemical and physical properties. Also, manganese oxides were reported as useful, adaptable, and ecofriendly catalysts for significant reactions and have been used widely for the conversion of a variety of molecules, especially for NO_x_ reduction [16], epoxidation of olefins [17], N-Alkylation of sulfonamides [18], ozone decomposition [19], oxidation of water [20] and benzyl alcohol [21], and for MRI contrast agent [22].

Additionally, the stabilization of catalyst is another main factor for obtaining reusable catalyst [11]. Some traditional stabilization approaches and techniques have been developed to obtain highly stable and reusable catalysts such as coating catalyst [23,24].

Lignosulfonate is a natural and aromatic biomaterial obtained as the waste by-product from pulping process and paper manufacturing [25]. Therefore, using lignosulfonate as a precursor for additional superior applications is in the center of attention in recent years [26]. Modrzejewska and coworkers used lignosulfonates as inexpensive and green stabilizer in their research [27]. Although how costly the novel metal has been reported in the applications of catalytic epoxidation [9], the nano MnO coated with calcium lignosulfonates as the sustainable and reusable catalyst in oxidation of styrene has not been reported yet.

Due to using hydrogen peroxide as oxidant, hydrophilic nanoparticles as the catalyst have attracted considerable attention for its chemical stability in aqueous media. As reported in the articles, products derived from styrene oxidation are highly diverse due to the type of catalyst and reaction conditions. Compounds such as acetophenone, benzyl alcohol, benzaldehyde, and benzoic acid are among the major products that can be produced in general in this oxidation. Although the conversion of styrene is sometimes over 90%, it is problematic to achieve the ideal product, and, in most cases, the production of lateral products would undermine the utility of the method [28].

**Scheme 1:**
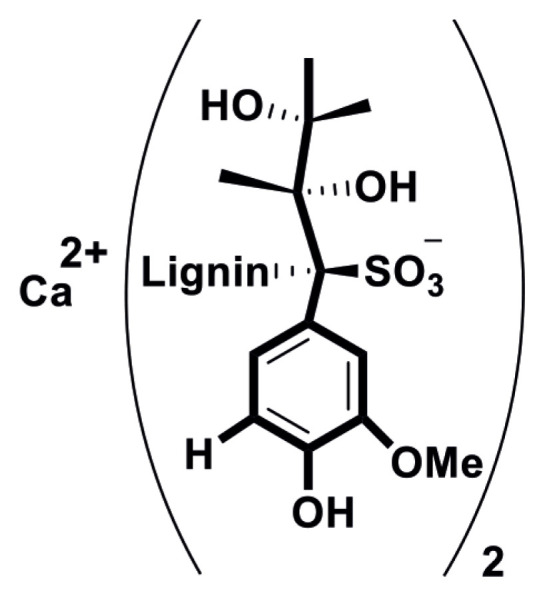
Chemical structure of calcium lignosulfonate.

**Scheme 2:**
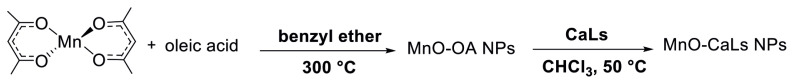
Reaction scheme for synthesis of nano catalyst.

Herein, we describe a facile and common process for phase transfer and modification of hydrophilic nanoparticles using calcium lignosulfonate as hydrophilic structure (Scheme 1). We have synthesized and characterized nano MnO-OA (manganese oxide nanoparticles capped with oleic acid) and stabilization of this metal oxide in aqueous media by exchanging its coating with calcium lignosulfonate (Scheme 2). This catalyst has been characterized by using various physico-chemical techniques like UV–vis, FT-IR, XRD, TG/DTA, AAS, and FE-SEM. Catalytic activity of MnO-CaLs towards oxidation of styrene was examined. To exhibit the utility of our designed catalyst, we employed hydrophilic catalyst for epoxidation styrene as the model.

One of the most important issues in this paper is to determine the optimum temperature and time conditions for the production of styrene epoxide with the negligible side product.

## 2. Experimental

### 2.1. Materials and methods

The chemicals used in this study can be listed as manganese (II) acetylacetonate (Sigma-Aldrich), benzyl ether (Sigma–Aldrich; 98%), styrene (Sigma-Aldrich), oleic acid (Merck), calcium lignosulfonate (Sigma–Aldrich). All common chemical materials and analytical grade solvents were used without further purification.

### 2.2. Catalyst preparation

#### 2.2.1. Synthesis of MnO-OA NPs

Mn(acac)_2_ as precursor (4 mmol) and oleic acid as a surfactant (4 mL) were dissolved in benzyl ether (40 mL) as a solvent. The mixture was degassed and stirred at the same time at 100 °C for 30 min to remove any moisture and oxygen. The mixture of reaction was quickly heated to 300 °C and refluxed at for 1 h under the argon atmosphere using a standard Schlenk line. By completing the reaction, color of the solution gradually changed to deep green. After completed the reaction, the mixture of the reaction was cooled to the room temperature, and then 200 mL of ethanol was added to reaction mixture. The precipitated NPs was collected by centrifugation (4000 rpm, 10 min) and washed by the mixture of acetone, ethanol, and n-hexane (1:1:1) three times. Finally, the purified manganese oxide nanoparticles with the oleic acid coating (MnO-OA NPs) were dispersed in chloroform for storage.

#### 2.2.2. Exchange MnO-OA to MnO-CaLs

**Scheme 3:**
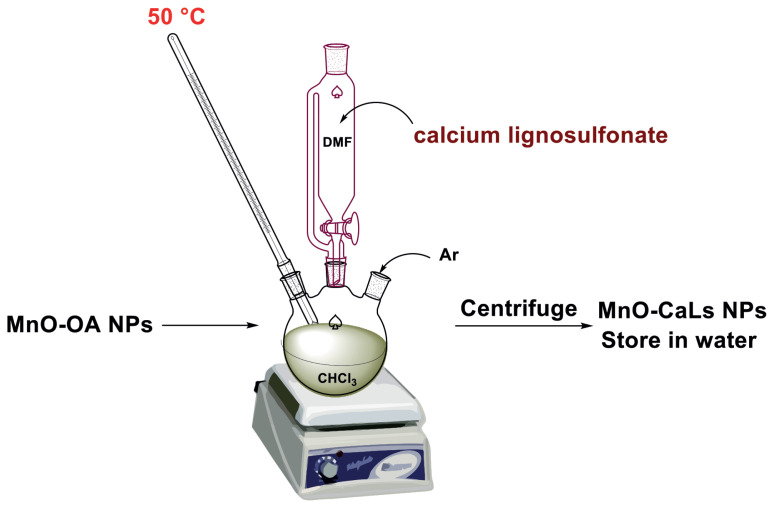
Reaction scheme for synthesis MnO-CaLs.

Synthesized hydrophobic MnO-OA NPs (100 mg) were dispersed in 100 mL of chloroform in a three-necked flask and then sonicated about 10 min until all nano particles dispersed completely. Separately, CaLs with average molecular weight of MW = 18000 (400 mg) was dissolved in DMSO (100 mL) and added dropwise to the mixture of the reaction (Scheme 3). The ligand exchange process was completed by stirring at 50 °C for 4 h under an inert atmosphere. The MnO-CaLs were collected by centrifugation (4000 rpm, 10 min) and then washed with the mixture of acetone and hexane three times. Finally, hydrophilic MnO-CaLs NPs were washed with ethanol and dispersed in ultrapure water.

### 2.3. Characterization of catalysts

**Figure 1:**
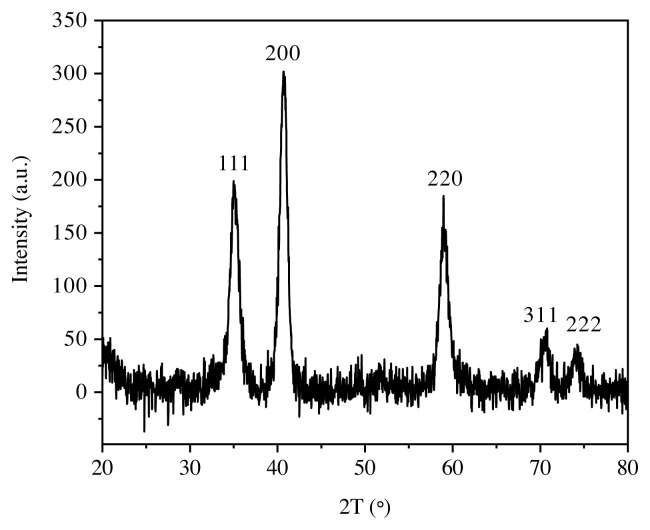
XRD pattern of MnO-OA NPs.

The XRD pattern is a unique feature of a catalyst, which is similar to fingerprinting, and the main method for determining catalyst phases [29]. Powder X-ray diffraction (XRD) patterns were used to analyze the crystallinity of the solid precipitates gained and to determine the MnO structures. The XRD patterns were obtained using a pw 3710 X-ray diffractometer (PHILIPS) equipped with a CuKα radiation (1.54442 Å). Figure 1 shows XRD patterns of the catalyst prepared; peaks of (111), (200), (220), (311), and (222) can be indexed to a cubic structure of MnO without other manganese oxide phases. According to the Debye–Scherrer equation, the crystallite size of MnO NPs was calculated » 9 nm.

Field emission scanning electron microscopy (FESEM) images were taken with a MIRA3-XMU microscope (TESCAN) at the operating voltage of 15 kV, and transmission electron micrography (TEM) images were taken with a PHILIPS CM30 instrument with an accelerating voltage of 150 kV. Preparing samples for TEM were done by dropping the nano particles scatterings on a carbon-coated copper grid and then evaporating the solvent under vacuum.

**Figure 2:**
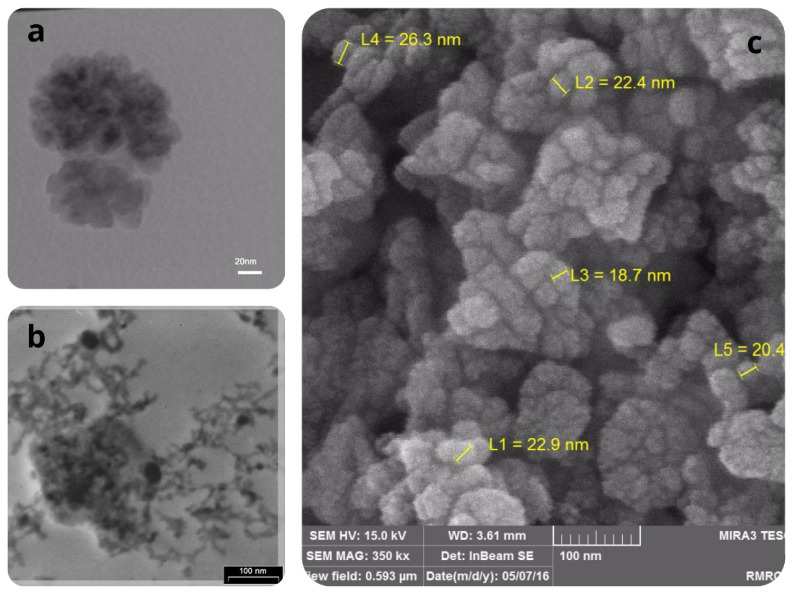
TEM images of a) hydrophobic MnO-OA NPs, b) hydrophilic MnO-CaLs NPs (manganese oxide nanoparticles stuck in calcium lignosulfonate polymeric network), and c) FESEM image of MnO-OA NPs.

The scanning electron microscope, as a powerful tool, provides the ability to study the crystalline structure of catalysts on a nanoscale, which most researchers use in the field of catalysts [30]. Figure 2a, derived from TEM, clearly demonstrates the fact that MnO-OA NPs has a good ability to phase change due to the proper and regular crystal structure. On the other hand, according to observations from this image, it can certainly be said that oleic acid completely covers all the metal oxide nuclei. This could emphasize the correct synthesis of the catalyst. In the Figure 2a, due to the fact that X-ray beam is passing through low density material, the dense material appears darker, and the low-density material appears lighter gray. According to the TEM image, the dark parts are related to manganese oxide, and the light parts are related to oleic acid, and, by using the image processing technique, the dark parts have an area of 45.68% of the total volume, so the remaining area, which is 54.32%, is related to oleic acid. Comparing TEM image and FESEM image of MnO NPs before and after phase transfer reveals that, by exchanging the ligand oleic acid with CaLs, these particles are stayed in highly-dispersed status without obvious aggregation. Also, Zhou et al. studied the crystalline state of the catalyst using the images obtained from SEM. The TEM image of MnO-CaLs was displayed after the phase transfer, MnO NPs that were attached to the polymeric structure of CaLs were separated from each other (Figure 2b). FESEM image of MnO NPs shows that nano particles before phase transfer are composed of 20–60 nm collectives of several 8–12 nm cores (Figure 2c).

**Figure 3:**
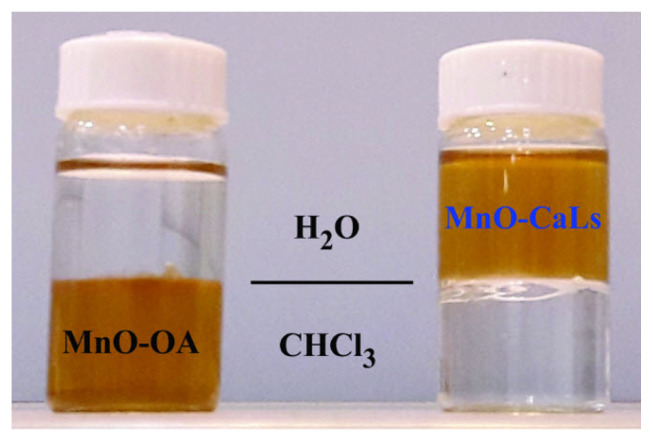
Solvent dispersity of nano MnO before and after phase transfer.

As shown in Figure 3, solvent dispersity of MnO shows that nano MnO-OA is dispersible in CHCl,_3_ and, by ligand exchanging, it has reasonable dispersible stability in water. After preparation, nano MnO-CaLs was stored in an aqueous media for two months and as a result, it shows good colloidal stability without aggregation in suspension. The colloidal stability can be related to the hydrophilic nature of calcium lignosulfonate and its strong bonding with nano MnO.

**Figure 4:**
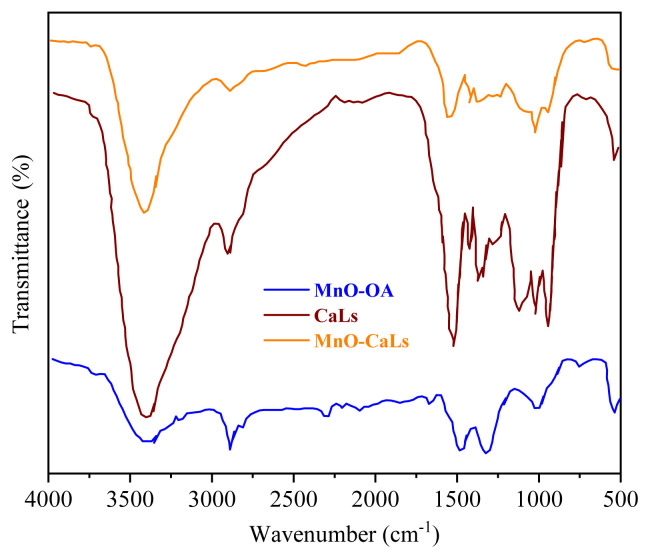
FT-IR spectra of MnO-OA NPs, MnO-CaLs, and calcium lignosulfonate.

Fourier transform infrared (FT-IR) spectra of the samples were obtained in the range of 400–4000 cm^−1^ with powders dispersed in KBr on Spectrum 65 FT-IR spectrometer (PerkinElmer). Today, infrared spectroscopy is widely used as an effective tool for identifying functional groups in chemical compounds [31]. Identification of the exchange of oleic acid with CaLs can be indirectly reflected by the FT-IR spectra information of samples. The FT-IR spectra of MnO-OA, MnO-CaLs, and CaLs samples are shown in Figure 4. The broad bands of MnO-OA at 2920 and 2855 cm^−1^ are attributed to the asymmetric and symmetrical stretching vibrations of the CH_2_ bands of oleic acid, respectively. A pair of bands in 1550 and 1410 cm^−1^ stand for symmetric and asymmetric stretching of the carboxylate group (COO^−^) of oleic acid [32]. Additionally, compared with MnO-CaLs NPs, it is worth noticing that these two bands disappeared. MnO-CaLs NPs exhibit absorbed band at 1460, 1514, and 1602 cm^−1^, which could be attributed to the characteristic peaks of aromatic rings. Also, the absorbance at 3420 cm^−1^ refers to the hydroxyl (O-H) stretching vibration. Additionally, the absorption peak shows an increasing intensity of OH band due to exchanging of oleic acid by CaLs. Furthermore, in all samples, the absorption bands at 520 and 655 cm^−1^ were assigned to MnO stretching vibrations.

**Figure 5:**
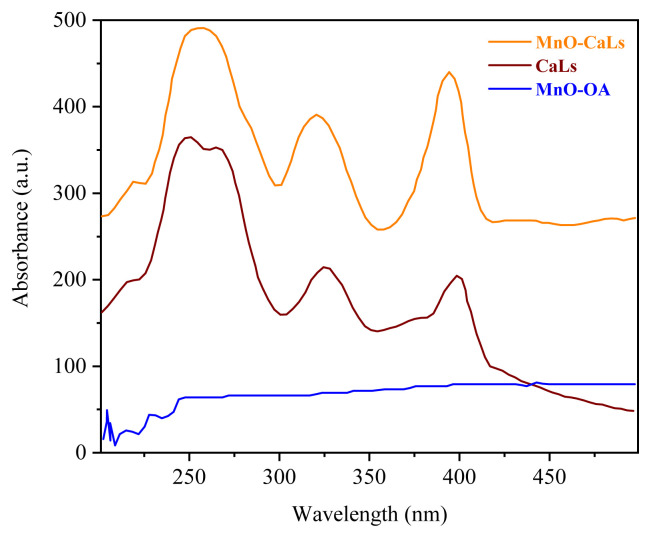
UV spectra of MnO-OA NPs, MnO-CaLs, and calcium lignosulfonate.

Diffuse reflectance UV-vis spectra of samples were obtained in the range of 200–400 nm by Lambda 35 UV-VIS Spectrometer (PerkinElmer). Afsar et al. [12] utilized UV spectroscopy in a catalytic process, which is an accepted method among researchers. To confirm the attachment of CaLs on MnO and replaced with oleic acid, the diffuse reflectance UV-Vis spectra is performed, and the results are shown in Figure 5.

**Figure 6:**
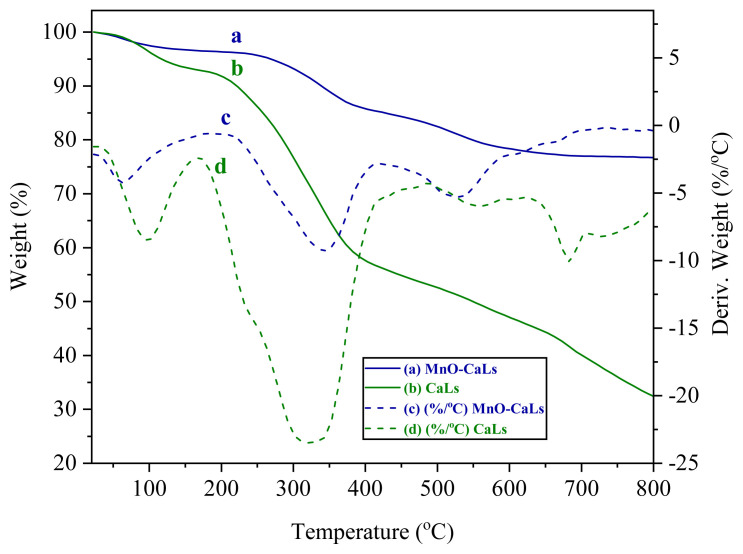
Thermal gravimetric analysis (TGA) and derivative thermogravimetry (DTG) of manganese oxide nanoparticles coated with calcium lignosulfonate (MnO-CaLs NPs) and calcium lignosulfonate (CaLs).

Thermal gravimetric analysis (TGA) and derivative thermal gravimetric (DTG) were performed with using TG 209 F1 Iris thermogravimetric analyzer (NETZSCH) with temperature varying from room temperature to 900 °C in a nitrogen atmosphere at a heating rate of 20 °C/min. TGA of the catalyst showed that the catalyst has good thermal stability (dec > 250 °C) (Figure 6).

For a deeper understanding of the thermal behavior of the synthesized catalyst, the curves for TGA are presented in a diagram for CaLs and MnO-CaLs. For this purpose, the values of TGA related to MnO-CaLs and CaLs are shown in Figures 6(a) and 6(b), respectively. The specific points in the graph where the slope change indicate the decomposition points of the sample. The differential weight loss ratio relative to the temperature causes these points to be better for thermal decomposition. Figures 6(c) and 6(d) refer to the above referrals. With accuracy in two DTG diagrams of MnO-CaLs and CaLs, we find that, at temperatures below 100 °C, a decomposition occurs in both samples, which refers to the evaporation of water absorbed at the surface of the samples. In the next step, the heat dissipation, which is accompanied by a more severe drop in the sample weight, maintains the sample MnO-CaLs to a higher temperature than the CaLs, and its weight loss is far less. The sum of the curves was provided in this diagram, and the synthesized catalyst in the temperature range of its application in the epoxidation process has excellent thermal stability.

The concentration of manganese ion in the prepared catalysts was determined using inductively coupled plasma optical emission spectroscopy by Spectro Arcos ICP-OES spectrometer.

## 3. Results and discussion

### 3.1. Oxidation of styrene

**Scheme 4:**
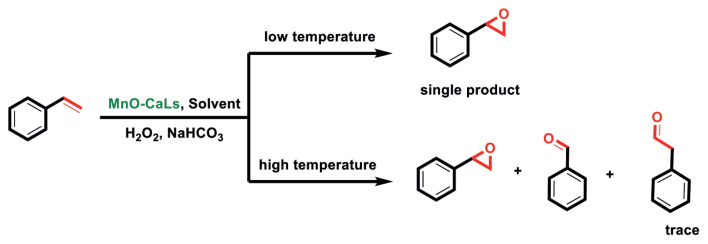
The route of selective oxidation of styrene.

Oxidation of styrene was carried out using MnO-CaLs as catalysts in the presence of H_2_O_2_ and NaHCO_3_. Due to its ease of use, hydrogen peroxide is considered to be a very suitable compound for the epoxidation of organic compounds [10]. Oxidation of styrene gives three oxidation products styrene epoxide, benzaldehyde, and phenylacetaldehyde (Scheme 4). These products are common and also have been identified recently [29]. Though, styrene epoxide and benzaldehyde were identified as the major oxidation products in the present work and selectivity of styrene epoxide can be increased by adjustment temperature.

Generally, MnO-CaLs (0.05g) and styrene (0.31 g, 5 mmol) were added into 10 mL of acetonitrile in a 50 mL round-bottom flask and stirred for 15 min and then the mixture of H_2_O_2_ (30%, 15 mmol) and NaHCO_3_ (0.2 mol L^−1^, 10 mL) was added dropwise into the solution within 0.5 h under continuous stirring (Scheme 2). After completion of the reaction, the nano catalyst was separated by centrifugation, and the mixture of the reaction was extracted by diethyl ether. The quantities of the obtained products were analyzed by GC, and the type of material was determined by matching the output materials of the GC with the library of the machine.

Product selectivity in various conditions is presented in Table. As it is clear, the best solvent for oxidation of styrene is acetonitrile and with increasing temperature from 0 to 70, the conversion rate of styrene clearly increases from 78% to 84%, but the selectivity of styrene epoxide decreases which is referred to producing byproducts and mainly conversion of styrene epoxide to benzaldehyde. These results indicate that temperature has a key role in the conversion of styrene oxidation and products selectivity. On the other hand, when reaction was carried out over 70 °C, styrene conversion completed, but the selectivity of styrene epoxide decreases due to the polymerization of styrene [33].

### 3.2. Effect of time, temperature, and amount of catalyst on selectivity of styrene epoxide

**Figure 7:**
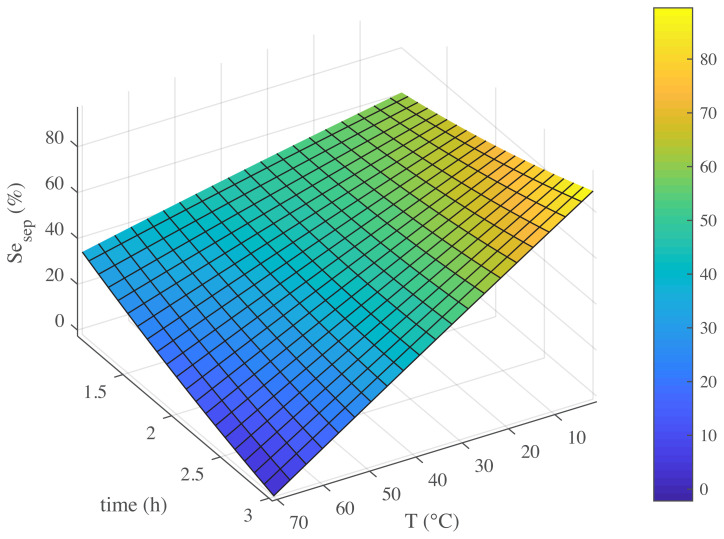
Effect of temperature and time on selectivity of Styrene epoxide (Se _sep_).

**Figure 8:**
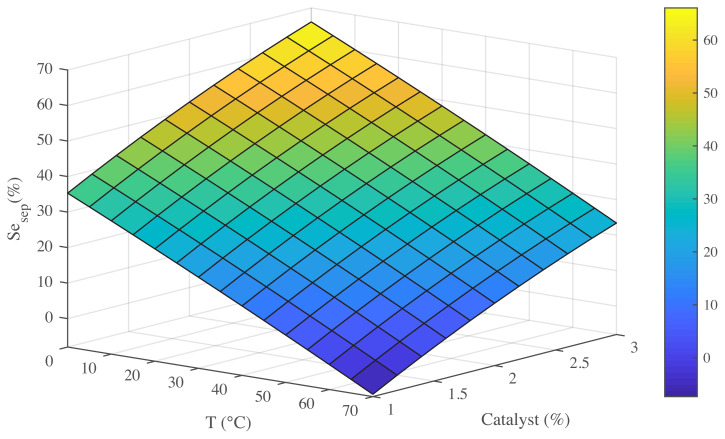
Effect of temperature and amount of catalyst on selectivity of styrene epoxide (Se _sep_).

**Figure 9:**
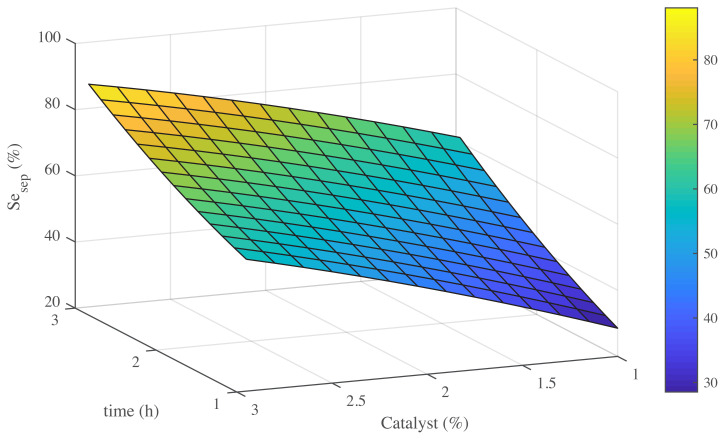
Effect of time and amount of catalyst on selectivity of styrene epoxide (Se _sep_).

To fully understand the influence of effective conditions on main product selectivity, it is important to determine all the variables. Furthermore, showing the response by 3D modelling can determine effects of all variables [34]. Exploration of these effects give a strong evidence for the reaction [35,36]. The effects of time, temperature, and amount of catalyst on the selectivity of styrene epoxide are presented in Figures 7–9.

It is seen from Figure 7 that, with the increase in temperature, the selectivity of styrene epoxide decreases due to increase in temperature oxidation of styrene epoxide, which might be occurred by the nucleophilic attack of H_2_O_2_ to styrene epoxide. According to 3D modelling, temperature and time have suitable effects but at closer look at this figure leads to the supposition that maximum amount of styrene epoxide formation is observed at 0 °C. In other words, it can be stated that the process behavior at the high and low temperatures is quite opposite. This contradiction in performance is clearly visible in Figure 7.

In this simultaneous form, the two parameters of temperature and time have changed as the most effective components in one shape, and their effect on product selectivity is drawn on the vertical axis. Because of the critical importance of temperature and time in the kinetics of the process, many researchers have examined the effect of these two parameters on various catalytic syntheses [3,37]. On this basis, between 1 and 3 h, temperature values have changed from 0 to 70 °C, which has changed from 0 to 100 °C in the selectivity. At high temperatures over time, the product reaches 0 °C. Zhou et al. [37] reported a similar process for an epoxidation process using a catalyst in their lower operating temperature. In this sense, based on their experiments, at a low temperature range and with a constant amount of a catalyst, at a similar temperature with more time, more products were obtained. Ma et al. [3] also presented a roughly similar process in terms of temperature and time effects at low temperatures for the amount of products in a heterogeneous catalytic epoxidation.

Gas chromatograph analyzes accurately states that at low temperatures, as in the upper branch of schematic 4, only styrene epoxide reaction occurs. However, at high temperatures, the results of analyzes include the production of two other side product, malononitrile and 2-phenylacetaldehyde. Side product manufacturing is generally considered to be unavoidable, but in some cases, it can be minimized by changing the reaction conditions [8,29]. From the side products, 2-phenylacetaldehyde is found to be in a slight amount. At high temperatures, the reaction progress like in the lower branch of schematic 4. The passage of time leads to a reaction pathway causes side products production. Meanwhile, production of malononitrile will be the dominant response. The recent reaction will be dominated by high temperature and long time at the end of the reaction, and no styrene epoxide will be observed.

Conclude what is described and can be seen in Figure 7 that the advantages of this catalyst are that unlike catalysts reported [3] so far for this process, it has the ability to produce Styrene epoxide with proper efficiency at low temperatures.

The same effect for temperature in various amounts of catalyst can be observed in Figure 8 and this figure demonstrates that the temperature plays the key role in Styrene epoxidation. The selectivity of Styrene epoxide was increased with the declining temperature in the range of 0–70 °C, and the amount of catalyst had changed from 1 to 3% in the reaction medium. In fact, this figure represents the interaction between reaction temperature and catalyst percent. Changes in catalyst content and its effect on the product are of interest to researchers working on organic synthesis [3,29]. What is important for the interaction of temperature and catalyst in the short term is that increasing the catalyst content is due to the rapid production of lateral products for the benefit of the main and desired reaction product, which is styrene epoxide. In this way, increasing catalyst content increases styrene epoxide selectivity by reducing the the production speed of other side products to an acceptable level. Also, the temperature increase somewhat improves this procedure, and, in low to high catalyst amounts, the process is roughly the same. In this way, as seen in Figure 8, the best conditions for increasing the production of the main product are the low temperature and the maximum amount of catalyst. Peng et al. [10], using a feed like our feed, in a catalytic epoxidation, expressed the effects of increasing the amount of reaction catalyst as expressed. Figure 8 also proves that the excellent properties of the catalyst nano MnO-CaLs have outstanding performance at low temperatures.

Figure 9 shows in various times by increasing amount of catalyst, product selectivity increased, and it is observed that controlling of amount of catalyst is the main factor for reaching the styrene epoxide. Especially, the maximum selectivity of styrene epoxide is located at the high amount of catalysis in the long run. This matter reveals the rules of time and catalyst on product selectivity. In detailed studies of the efficiency of catalysts, researchers often want to change the amounts of catalyst present in reaction and process time [2,21,28]. In principle, Figure 9 contains the interaction of two important parameters, namely time and catalyst percentages. It is noteworthy that it should be noted that Figure 9 is drawn from a catalyst performance for a low temperature, not for high temperatures. The reason for choosing the low temperature in harmony with the description of Figure 8 is that it is debatable that at high temperatures, the reaction to the production of the side products will be dominant and the optimal product, which is the same styrene epoxide, is produced at low temperatures. As seen in Figure 9, the time ranges from 1 to 2 h, and the nano MnO-CaLs catalyst content varies between 1 and 3%. In this figure, with increasing temperature, the styrene epoxide selectivity increases, although this process tends to increase strongly in higher concentrations of nano MnO-CaLs catalyst. The highest amount of the desired product after 2 h is observed at a concentration of 3% of the catalyst. In agreement with what was discussed about the effect of temperature, Vieira et al. [2] reported a similar process for the epoxidation reaction using a heterogeneous catalyst.

### 3.3. Recyclability of the catalyst

**Figure 10:**
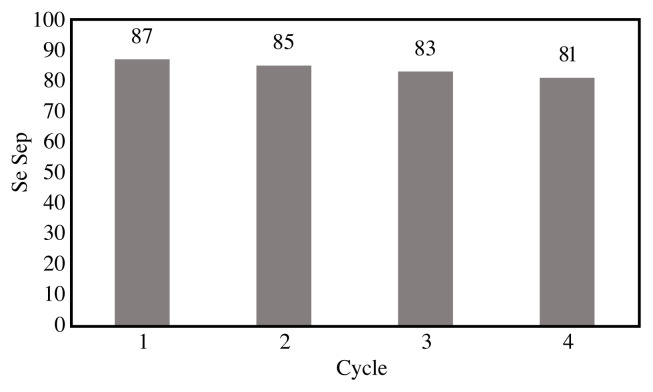
Recycle of catalysis.

The recyclability of catalyst is one of the crucial factors for a prominent heterogeneous catalyst from economic and environmental points of view. Thus, the recyclability of nano MnO-CaLs catalyst was investigated under the optimized reaction conditions towards the selective oxidation of styrene. This catalytic system could be simply recovered from the mixture of reaction by centrifugation and subjected to the next run. What is certain is that the catalyst recycling study is in fact the most intuitive tool for determining catalyst activity [10]. This means that increasing the selectivity of the desired product after each catalyst recycling involves its high activity. As summarized in Figure 10, the conversion of styrene was slightly reduced (< 10%). According to this figure, even after four times the recovery, catalyst activity is still within acceptable limits.

## 4. Conclusion

In summary, we have reported that nano structure of MnO-CaLs acts as an efficient hydrophilic catalyst for catalytic oxidation of styrene. MnO-CaLs with reasonable stability, nano structure and environmentally friendly were synthesized at inert atmosphere. The process could be made selective towards the formation of styrene epoxide when employing acetonitrile as solvent at 0 °C or favor the formation of benzaldehyde by increasing temperature and using an excess amount of H_2_O_2_.

## Figures and Tables

**Table:** product selectivity of oxidation of Styrene^a^.

Entry	T (°C)	catalyst	Solvent	Conversion (%)	Styrene epoxide^b^ (%)	Benzaldehyde^c^ (%)
1	0	MnO-CaLs	H_2_O	50	80	-
2	0	MnO-CaLs	DMF	70	75	-
3	0	MnO-OA	CH_3_CN	7	98	-
4	0	-	CH_3_CN	-	-	-
5	0	MnO-CaLs	CH_3_CN	78	84	-
6	25	MnO-CaLs	CH_3_CN	80	60	
7	50	MnO-CaLs	CH_3_CN	80	30	60
8	70	MnO-CaLs	CH_3_CN	84	-	70
9	80	MnO-CaLs	CH_3_CN	100	-	-

a Product selectivity in various conditions at the 3rd h.

b Selectivity of styrene epoxide.

c Selectivity of benzaldehyde.
